# Static and dynamic intracerebral signal analysis reveals protective networks against seizures in drug-resistant focal epilepsy

**DOI:** 10.1093/braincomms/fcag047

**Published:** 2026-02-20

**Authors:** Roberta Di Giacomo, Pablo Núñez, Jesús Poza, Victor Rodríguez-González, Carlos Gómez, Alessandra Burini, Laura Castana, Marco de Curtis, Laura Tassi, Giulia Varotto

**Affiliations:** Epilepsy Unit, Fondazione IRCCS Istituto Neurologico Carlo Besta, Milan 20133, Italy; Coma Science Group, GIGA-Consciousness, University of Liège, Liège 4000, Belgium; Biomedical Engineering Group, University of Valladolid, Valladolid 47011, Spain; Centro de Investigación Biomédica en Red de Bioingeniería, Biomateriales y Nanomedicina (CIBER-BBN), Madrid 28029, Spain; Biomedical Engineering Group, University of Valladolid, Valladolid 47011, Spain; Centro de Investigación Biomédica en Red de Bioingeniería, Biomateriales y Nanomedicina (CIBER-BBN), Madrid 28029, Spain; IMUVA, Instituto de Investigación en Matemáticas, University of Valladolid, Valladolid 47011, Spain; Instituto de Investigación Biosanitaria de Valladolid (IBioVALL), Valladolid 47010, Spain; Biomedical Engineering Group, University of Valladolid, Valladolid 47011, Spain; Centro de Investigación Biomédica en Red de Bioingeniería, Biomateriales y Nanomedicina (CIBER-BBN), Madrid 28029, Spain; Instituto de Investigación Biosanitaria de Valladolid (IBioVALL), Valladolid 47010, Spain; Biomedical Engineering Group, University of Valladolid, Valladolid 47011, Spain; Centro de Investigación Biomédica en Red de Bioingeniería, Biomateriales y Nanomedicina (CIBER-BBN), Madrid 28029, Spain; Instituto de Investigación Biosanitaria de Valladolid (IBioVALL), Valladolid 47010, Spain; Epilepsy Unit, Fondazione IRCCS Istituto Neurologico Carlo Besta, Milan 20133, Italy; Department of Medicine (DMED), Neurology Unit, University of Udine, Udine 33100, Italy; Claudio Munari Epilepsy Surgery Centre, Niguarda Hospital, Milan 20162, Italy; Epilepsy Unit, Fondazione IRCCS Istituto Neurologico Carlo Besta, Milan 20133, Italy; Claudio Munari Epilepsy Surgery Centre, Niguarda Hospital, Milan 20162, Italy; Epilepsy Unit, Fondazione IRCCS Istituto Neurologico Carlo Besta, Milan 20133, Italy; Laboratory for Clinical Neuroscience, Center for Biomedical Technology, Universidad Politécnica de Madrid, Madrid 28223, Spain

**Keywords:** drug-resistant epilepsy, stereo-electroencephalography, brain networks, functional connectivity, epileptogenic zone

## Abstract

Epilepsy research increasingly emphasizes the role of brain network dynamics in seizure generation and propagation. Nevertheless, the interplay between the mechanisms that enhance or inhibit seizure initiation remains poorly understood. In this study, we explore both static and dynamic functional brain networks preceding ictal minor electrical discharges and major seizures, within epileptogenic and non-epileptogenic zones, using intracerebral recordings from patients with drug-resistant focal epilepsy. Stereo-electroencephalographic signals were recorded from 39 patients with focal drug-resistant epilepsy during pre-surgical monitoring. Static functional connectivity was quantified using graph theory metrics, whereas dynamic connectivity was characterized through the analysis of the complexity and dwell times of brain meta-state activations. Static connectivity analysis revealed significant alterations in network centrality, integration, and segregation properties, with distinct patterns characterizing resting conditions, minor electrical discharges and major seizures. Specifically, network analysis before minor electrical discharges exhibited increased nodal strength and reduced betweenness centrality in the epileptogenic zone, associated with greater integration and reduced segregation in non-epileptogenic zones. Dynamic connectivity analysis showed lower complexity and longer stability of meta-states before minor electrical discharges, particularly in high-frequency signals of non-epileptogenic zones. Taken together, our findings provide novel and valuable insights into the topological organization and the dynamic changes of brain networks before epileptic seizures, suggesting the presence of a protective mechanism, mainly involving non-epileptogenic zones, able to prevent minor electrical discharges to evolve into a mayor seizure. A better understanding of these network changes is pivotal for improving therapeutic strategies in epilepsy, particularly those targeting dynamic network alterations.

## Teaser

Di Giacomo *et al*. applied static and dynamic functional connectivity analysis to stereo electroencephalography (SEEG) in drug-resistant epilepsy, investigating differences in networks preceding minor electrical discharges and major seizures. Results reveal regulatory mechanisms, mainly involving regions beyond the epileptogenic zone, which may prevent minor electrical discharges from evolving into full seizures.

## Graphical Abstract

**Graphical Abstract:**
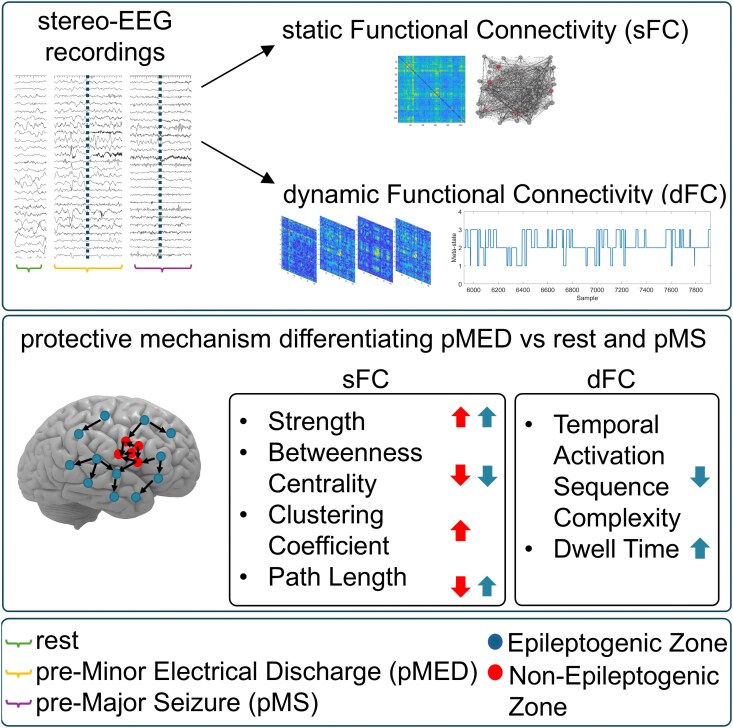

## Introduction

The unpredictability of the appearance and recurrence of seizures leads to significant impact in the life of people with epilepsy. Understanding pre-ictal electrophysiological alterations could offer valuable information to better understand seizure-related mechanisms, and to develop proper tools for seizure prediction aimed at controlling seizure occurrence. Human brain functions are increasingly understood and explained in terms of large-scale complex brain networks that dynamically evolve across multiple spatial and temporal scales. Epilepsy and several others neurological diseases have been associated with abnormal modifications of these networks.^1,2^ However, how these alterations reorganize before seizure onset and determine whether an event remains subclinical or evolves into a full seizure remains largely unknown despite its potential clinical relevance for localizing the epileptogenic zone (EZ) and predicting seizure occurrence.

Despite the large body of studies on functional connectivity (FC) in epilepsy, the understanding of the mechanisms ruling epileptogenic networks remains elusive.^3^ Most of these studies focus on *static functional connectivity* (sFC), which assumes temporal stationarity of the statistical interdependencies between signals recorded in different brain regions, thus ignoring the temporal evolution of these networks. As neuronal oscillations evolve very quickly in time,^4^ FC also reflects these rapid changes, exhibiting a high dynamic variability in time and space.^5^ The field of *dynamic functional connectivity* (dFC)^1,6^ paved the way to assess the fast fluctuations of neural networks, allowing to take full advantage of the high temporal resolution of electrophysiological data. However, only a few intracranial EEG studies have applied dFC frameworks to explore pre-ictal network dynamics, and none, to our knowledge, have examined their role in distinguishing minor electrical discharges (MED) from major seizures (MS).

In this study, we used a comprehensive approach encompassing both sFC and dFC analyses. First, we used graph theory methods to quantify the topological sFC differences between different conditions. Second, we applied a dFC-based methodology to detect brain network configurations recurrent over time (referred to as ‘meta-states’) that act as attractors of the neural network.^7,8^ Recently, there has been a growing interest in the study of these recurrent brain states.^5,8-10^ This dynamic approach offers a novel and powerful framework to characterize how large-scale connectivity evolves during cognitive or pathological processes. In the field of epilepsy, functional magnetic resonance imaging (fMRI) was applied either to compare brain networks states in epileptic patients and healthy controls^11,12^ or to assess the effect of intracerebral electrical stimulation.^13^ Despite its wide use, fMRI signals have a reduced temporal resolution compared with electrophysiological data, which limits the analysis of fast brain dynamics. Conversely, electroencephalography and magnetoencephalography (MEG) capture physiological and pathophysiological activity on various time scales with a high temporal resolution, although requiring more sophisticated time-series-analysis techniques.^14^ The application of this dynamic framework to invasive SEEG recordings therefore provides a unique opportunity to bridge the temporal precision of electrophysiology with the network-level interpretability of dFC approaches.

The gold-standard procedure to identify the brain areas responsible for seizure generation in drug-resistant epileptic (DRE) patients is the stereo-electroencephalography (SEEG); this diagnostic method is utilized during pre-surgical evaluation to identify the cortical areas to be resected to cure the patients.^15,16^ These invasive intracerebral recordings are performed when no lesion is detected with neuroimaging techniques, or when the boundaries of the lesion cannot be clearly outlined.^16,17^ SEEG reveals interictal and ictal activities that, together with semiology and anatomical data, define the EZ, i.e. the cortical areas indispensable for generation and primary organization of the seizure discharge.^15,18,19^ Moreover, SEEG represents a unique tool to localize and identify interictal activities in the lesional zone (i.e. cortical area where background activity is altered and slow waves are predominant) and the irritative zone (i.e. site of spiking activity).

The EZ can generate full-blown seizures as well as MED.^20-23^ These discharges differ from the ongoing interictal background activity; visually, MED show an electrical pattern similar to the seizure onset generated in the same cortical area during major seizures (MS).^24,25^ Estimated using SEEG microelectrodes, MED have very short duration and minimal (if any) clinical correlates.^20^ MED can spread to the same regions of MS or they can involve different areas compared with MS. The co-localization of MED and MS correlates with the post-surgical outcome in patients who underwent resective surgery.^22-25^ Yet, the network mechanisms determining whether activity in the EZ remains confined (MED) or evolves into a seizure (MS) are still poorly understood.

Here, we address this critical gap by characterizing, for the first time, both static and dynamic intracerebral network reorganization preceding MED and MS using SEEG. Building on previous theoretical frameworks—such as the *interictal suppression hypothesis*^26-28^ which posits that the EZ is inhibited by other brain regions during interictal periods and that seizures emerge when this inhibitory control fails, and the *pull-push theory*^29,30^ which proposes a competitive interplay between synchronizing and desynchronizing regions that constrains seizure spread—we hypothesized the existence of a protective network mechanism that prevents the evolution of MED into MS, arising from topological and temporal reconfiguration of brain regions beyond the EZ. Starting from the evidence that the SEEG patterns of onset of MED and MS are similar, we considered MED as a suitable and novel model to disentangle mechanisms of seizure suppression versus facilitation. To this aim, we evaluated differences in the sFC and dFC of intracerebral networks preceding both MED and MS onsets and compared these to baseline activity during the interictal condition.

## Materials and methods

### Subjects

Patients with DRE undergoing long-term SEEG recordings during the pre-surgical work-up were retrospectively selected from the clinical database collected at the *Claudio Munari* Epilepsy Surgery Centre of the Niguarda Hospital in Milan, over a 17-year historical extension (2005–2022). Database collection for research purposes was approved by the local Ethics Committee and informed consent was signed by all patients. We selected patients with: (i) both MS and MED (ii) available recordings of a sixty-second epoch free from epileptiform discharges before these events; (iii) MS starting with a sequence of events similar to MED were included in the study and (iv) at least one-year of follow-up evaluated with Engel’s seizure outcome scale. We selected patients with EZ clearly defined based on clinical and SEEG data, in which surgery or radiofrequency SEEG-guided thermocoagulation was subsequently performed. Functional networks can be influenced by various biological rhythms, drugs and individual brain organization. We performed the analysis on the same patient, during waking period, under constant medication, to avoid the influence of long-term temporal or pharmacological dynamics.

### SEEG recording and preprocessing

**Figure 1:**
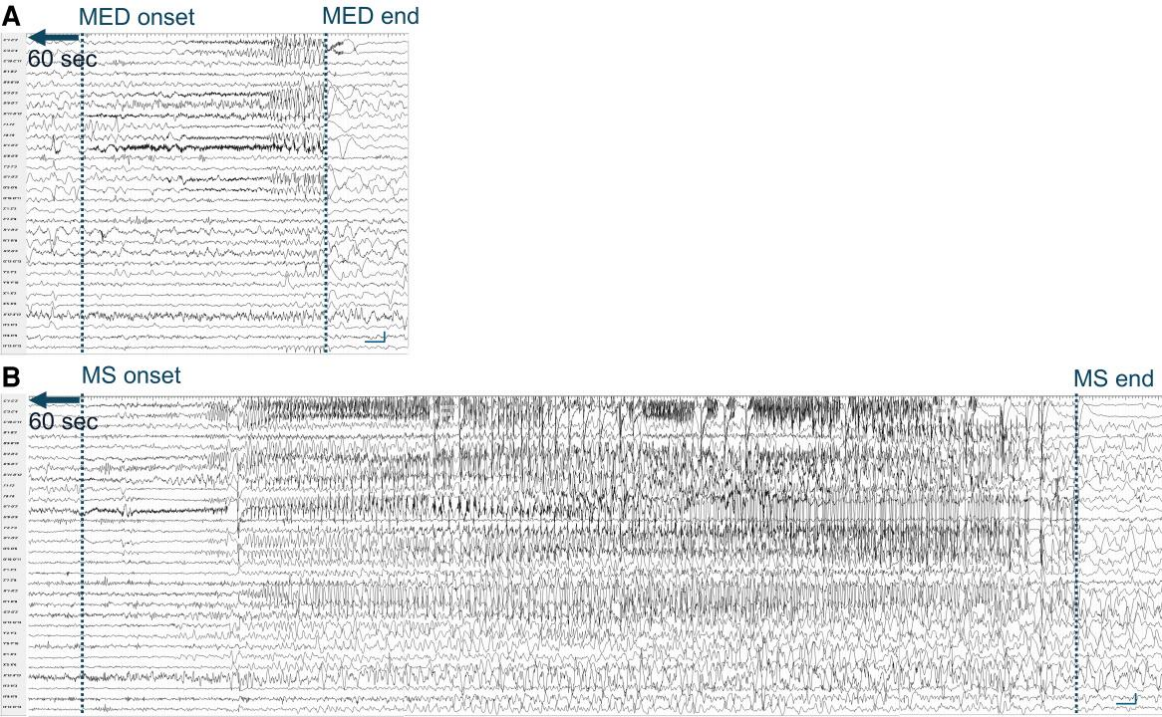
**Stereo-EEG recordings of minor electrical discharge (MED) and major seizure (MS) for a representative patient.** Both panels show a subset of 30 selected stereo-EEG contacts. (**A**) MED, lasting 12 s and involving mainly temporo-mesial, temporo-polar and insular regions. (**B**) MS from the same patient, lasting 62 s, involving the same contacts of MED and also orbito-frontal contacts. Calibration bars in stereo-EEG recordings: vertical line 100 µV, horizontal line 1 s.

For SEEG video-monitoring, a Nihon Kohden System with 192 channels recorded at a sampling rate of 1000 Hz was used. SEEG was tailored to the patients’ individual anatomic and electro-clinical characteristics.^16^ The electrode trajectory planning was optimized to cover the hypothesized EZ based on non-invasive data (MRI, PET, MEG, etc.) and to minimize the risk of underestimation. Despite meticulous planning, the possibility of subtle, unsampled epileptogenic tissue cannot be completely excluded, potentially impacting long-term outcome. This is the reason for our choice to select patients with good post-surgical outcomes, in whom EZ was clearly defined on SEEG (given that a poor surgical outcome is explained by incomplete resections of the measured SOZ due to its proximity to the eloquent cortex as well as other anatomical constraints). Conversely, seizure activity may spread rapidly from the true EZ to surrounding non-epileptogenic tissue, making it difficult to distinguish primary onset from secondary propagation. The potential for overestimation of the EZ/NEZ boundaries was addressed by using neurophysiological biomarker of focal ictogenesis^31,32^ and by a careful and systematic assessment of clinical correlates and cortical activity responsible for the onset and the primary organization of the seizures. Multi-contact electrodes with 5–18 recording sites (2 mm in length and 1.5 mm apart; Dixi; Besançon, France) were intracerebrally inserted according to the standard diagnostic protocol.^16^ Wake and sleep recordings were visually examined by two neurologists (R.D.G. and A.B.) to define the MED and MS onset and to delineate contacts included in the EZ. MEDs were not associated to obvious clinical manifestations and were identified as low-voltage fast activity discharges, often associated with very slow potentials that could gradually develop into a rhythmic theta-delta discharge (Fig. 1), followed by electrical depression and/or slow waves. Consensus on the identification of both MED and MS, and EZ boundaries was obtained between both professionals, solving the divergences with open discussions. Figure 1 illustrates representative SEEG signals recorded during MED and MS from one patient.

One MED and one MS were identified and analyzed for each patient. Sixty seconds preceding the onset of both MED (defined hereafter as pMED) and MS (pMS) during wake were selected for each patient. Moreover, for each subject, 60 s of resting state activity (rest) at least 10 min away from any type of ictal discharge were chosen as baseline condition. The initial selection of 60-s time segments for the dFC analyses was based on a previous study in which dFC pipeline was presented and successfully applied to resting-state EEG recordings.^7^ An extensive evaluation of the effect of segment length was also conducted, showing that a 60-s segment length was sufficient to properly extract brain meta-states and their sequencing.^33^ Contacts located within the white matter, identified through MRI and electrical activity, were excluded from the analysis. Contacts with artifacts were visually identified and excluded from the analysis. All SEEG signals were recorded and analyzed using a referential montage, preserving the full amplitude and phase information of each channel. This approach is standard in clinical practice and is particularly suitable for estimating amplitude-based connectivity measures such as amplitude envelope correlation (AEC) and instantaneous amplitude correlation (IAC).^34^ While the choice of reference electrode can influence connectivity metrics, especially by introducing spurious zero-lag correlations, we applied signal orthogonalization prior to connectivity estimation (as detailed in the following section). This procedure effectively removes zero-lag linear dependencies and minimizes the impact of reference-related and volume conduction artifacts. All recordings were down-sampled to 250 Hz to reduce computational requirements. It has been shown that epileptic dynamics manifest differently across frequency ranges. For instance, seizure onset is best described by low-voltage fast discharges in the beta and gamma frequencies,^15,31^ and EEG-based metrics benefit from band decomposition to differentiate between ictal and interictal EEG.^35^ To allow these granular analyses, the recordings were then filtered in the delta (1–4 Hz), theta (4–8 Hz), alpha (8–13 Hz), beta1 (13–19 Hz), beta2 (19–30 Hz), and gamma (30–48 Hz) bands by means of finite impulse response (FIR) filters, with both forward and backward filtering.

### Static functional connectivity

The sFC was estimated by means of the AEC.^36^ The orthogonalized AEC was selected due to its robustness against volume conduction effects, a major source of bias in connectivity analyses, and its proven reproducibility and consistency, both considered quality markers for connectivity metrics.^37,38^ Additionally, its mathematical simplicity facilitates interpretation compared with more complex measures such as phase locking value or coherence.^36,37^ Finally, as our dFC analyses employed the IAC, the dynamic counterpart of the AEC, it is methodologically consistent to rely on metrics based on similar principles. For each of the three conditions (rest, pMED, and pMS) sampled in each patient, the 60-s filtered SEEG signals were divided into 12 non-overlapping 5 s-length epochs. We used 5-s epochs because sFC metrics have been shown to remain stable at short epoch lengths, with minimal variance when epochs are further extended. Thus, 5-s epochs provide an optimal balance between stable statistical estimation and reliable feature extraction.^37,39,40^ The time series were then orthogonalized to minimize spurious correlations.^5^ Next, the AEC was calculated on each epoch and was averaged across them, thus obtaining an adjacency matrix of sFC for each subject and each of the three conditions. The size of the sFC matrices varied across patients, according to the number of SEEG contacts analyzed (number of contacts = 123.10 ± 26.82).

**Figure 2:**
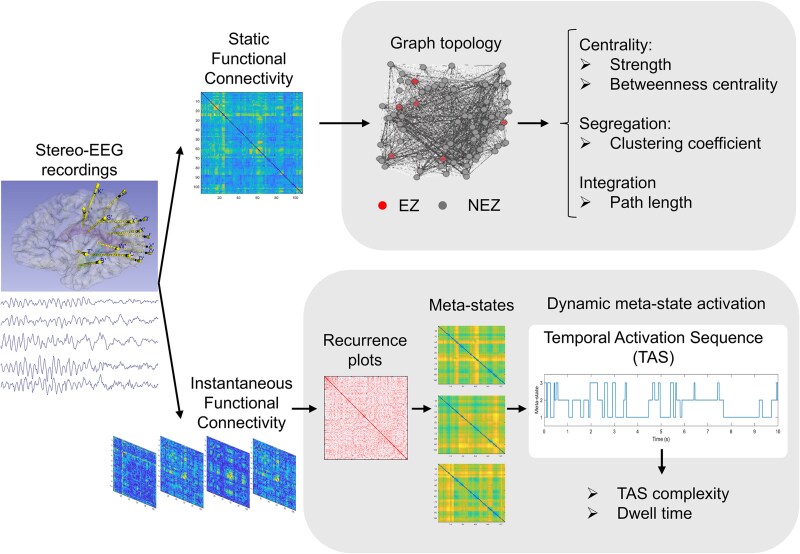
**Methodological workflow. Upper part—sFC.** The brain connectivity was estimated using a static approach by means of the orthogonalized AEC, and different network properties (centrality, segregation and integration) were assessed for both the EZ and NEZ regions. Lower part—dFC. The dFC was computed by: (i) calculating the instantaneous connectivity tensors by means of the IAC; (ii) computation of recurrence plots that assess recurrences in the connectivity matrices across time; (iii) identification of subject-based brain states recurrent in time, referred as ‘meta-states’; (iv) extraction of meta-state activation dynamics; and (v) estimation of dwell time and TAS complexity.

Connectivity matrices were represented through a graph, a mathematical object which allows to extract quantitative parameters to describe and summarize different topological properties of the network under study, such as centrality, integration, and segregation.^41^ A graph is defined by a set of nodes (in this case SEEG contacts) and weighted edges connecting the nodes (i.e. the value of AEC between each pair of contacts). The static network topology during rest, pMED, and pMS was described using graph theory indices that capture centrality, segregation, and integration (Fig. 2). Global connectivity was first assessed by the global strength, defined as the sum of all edges in the network and reflecting the overall level of interconnection among regions. To evaluate centrality, which reflects the importance of a node within the network, we employed strength (the sum of a node’s edges) and betweenness centrality (the extent to which a node lies on shortest paths between other nodes). Segregation, or the tendency of a network to form densely interconnected subgroups, was quantified using the clustering coefficient. Finally, integration, which reflects the network’s ability to combine information across regions, was assessed by the characteristic path length, defined as the average shortest path between any two nodes and indicating the efficiency of global information exchange. The selection of these graph measures was motivated by their widespread use as representative indices of brain network organization.^42-44^ While additional metrics exist for each property, we sought to provide a parsimonious and intuitive description of the networks. Accordingly, a single representative measure was chosen for each property (two in the case of centrality, given its relevance and complementarity). Although additional indices might offer further nuance, they often provide overlapping information and would be unlikely to alter the main conclusions. It is well known that the size of a network (i.e. the number of nodes and edges) can significantly influence its functional features.^45-47^ To mitigate this issue, we implemented two complementary strategies. First, all network metrics (except for global strength) were computed on graphs normalized to the same global strength (i.e. total network strength set to 1). This procedure ensured that differences in metric values were not merely driven by variations in overall connectivity levels, thereby allowing for meaningful comparisons across networks of different sizes and densities. Second, we applied a bootstrap downsampling approach inspired by previous studies.^45-49^ Specifically, the nodes of each graph were randomly reduced to the minimum common subset across patients, resulting in networks of 48 nodes (4 from the EZ, and 44 from the NEZ). To minimize the influence of random selection, this downsampling was repeated 100 times. For each repetition, all graph-theoretical metrics were computed, and the results were averaged across repetitions. Finally, local indices were averaged for each node within the EZ and NEZ regions of interest, ensuring that the reported metrics reflect robust estimates not influenced by differences in network size.

### Dynamic functional connectivity

The dynamic fluctuations of the network were assessed by means of a published method based on detecting recurrent network topologies over time, known as meta-states.^7^ These meta-states are considered as attractors of the neural network in each moment, governing their dynamic fluctuations across time. This methodology begins by computing the IAC, a high temporal resolution version of the AEC (see Tewarie *et al*., for method details).^9^ For each of the three conditions (rest, pMED, and pMS), band-passed filtered SEEG signals were utilized to calculate the IAC between each pair of SEEG contacts for each 60-s time sample. For each patient, we built a dynamic connectivity matrix of: [3 conditions × *N* contacts × *N* contacts × 15 000 temporal samples (60 s·250 Hz)], with the number of contacts being different for each subject. Of note, before computing the IAC, the time series were orthogonalized to minimize volume conduction effects.^5^

After calculating instantaneous FC using IAC, we explored the temporal recurrences of connectivity matrices (i.e. time slots in which the connectivity presents similar network topology) using recurrence plots (RPs), which are commonly used to visualize recurrent patterns in dynamical systems.^50^ The RPs were matrices of size 15 000 × 15 000, where each element *C_ij_* (with *i,j* = 1,…,15 000) indicates the correlation between the FC matrices in time samples *i* and *j*, calculated as the Spearman correlation between them. To reduce the computational cost of the methodology, we applied a data-driven windowing method^9^ that detects time periods where the functional connectivity matrices can be considered as stable. The IAC matrices were thus averaged in the time slots indicated by the above data-driven windows, and new (temporarily-constrained) RPs was constructed. This approach also presents the advantage of minimizing the effects of noise, as described in detail by Tewarie and colleagues.^9^

As a next step, we considered the RPs to be graphs, in order to be able to perform community detection on them, as follows: within each RP, the temporal axes (i.e. the data-driven windows) were considered to be nodes in a graph, and the nodes were connected to each other by the correlation between the functional connectivity of each window (i.e. the edges of the graph). Next, we applied the Louvain GJA community detection algorithm to the temporarily-constrained RPs to identify network topologies recurrent over time (i.e. communities),^51^ without *a-priori* defining the number of communities to detect. The Louvain GJA algorithm performs community segmentation maximizing modularity^42,52^ and reveals groups of time windows that are similar to others,^7^ The community detection was individually performed for each patient, as the different number and spatial locations of SEEG electrodes prevented the detection of group-representative states across subjects. To do this, we concatenated the windowed connectivity for the three conditions (rest, pMED, and pMS) to generate the RPs on which the Louvain GJA algorithm was applied. This allowed us to obtain the meta-states particular to each subject and their corresponding FC matrices.

Once the meta-states were obtained, we went back to the original, non-temporally aggregated connectivity matrices. Each temporal sample was assigned to a dominant meta-state by performing Spearman correlation of the FC matrix in that time sample with the FC topology of all the identified meta-states (i.e. the meta-state displaying higher correlation with the network in that time sample was determined to be dominant for that time point). The symbolic time series indicating the dominant meta-state (i.e. the assigned meta-state) in each sample during the 60-s epoch is called Temporal Activation Sequence (TAS).^7^ Derived from this metric, we employed two additional parameters:

#### Dwell time

This metric evaluates the average time the brain remains in the same dominant meta-state (Núñez *et al*.^58^). It is a widely used parameter in studies of dynamic brain state transitions, also known in the literature as ‘lifetime’.^53^

#### TAS complexity

To effectively capture the richness of the meta-state sequencing, we utilized Lempel-Ziv complexity (LZC).^7^ LZC relates to the number of distinct substrings and their rate of occurrence, with higher values indicating greater complexity.^54^ The LZC algorithm follows the method described by Abásolo *et al*.,^54^ with the only difference being that the TAS is already a finite symbol sequence, so no conversion is required.

Finally, the whole process was repeated after removing the contacts corresponding to the EZ, obtaining a NEZ network; and meta-states for each subject without EZ contacts, as well as their corresponding TAS and ICT, were computed. Due to the reduced number of contacts in EZ in some subjects, and the high complexity of these analyses, it was not possible to perform them exclusively on the EZ. The methodological procedure, including static and dynamic FC analyses, is illustrated in Fig. 2.

### Surrogate data for metric normalization

To determine whether the extracted measures represented genuine dFC rather than random fluctuations, we conducted surrogate data testing.^7,55^ To this end, we created surrogate versions of each EEG recording using the amplitude-adjusted Fourier transform (AAFT), a method that retains the amplitude distribution of the original time series.^56,57^ Importantly, we kept the same sequence of random numbers (uniform phase randomization) in all SEEG channels to maintain linear correlations and preserve static functional connectivity (FC).^55,57^

Following previous studies,^7,8,58^ all measures were normalized by dividing them by the average values calculated from 100 surrogate time series. This approach ensures that values close to 1 indicate behaviour that can be attributed to random fluctuations in sFC, while the further away from 1 the value is (either higher or lower), the more it reflects behaviour inherently associated with dFC.

### Statistical analysis

The same statistical procedure was applied for both static (strength for the global network; and strength, betweenness centrality, clustering coefficient, and characteristic path length for EZ and NEZ regions) and dynamic metrics (TAS complexity and dwell time, for the global and the NEZ network). To avoid the assumption of normality and homoscedasticity of data, the non-parametric Friedman test was first applied to detect statistical differences among rest, pMED, and pMS conditions, for all frequency bands. To control type I error, false discovery rate (FDR) correction was applied to control for the number of bands and metrics.^59^

When a significant interaction was present, a *post hoc* Wilcoxon signed rank test was performed to assess pairwise between-condition differences. A significance level of 0.05 was considered. Again, an FDR correction was used, so all *P*-values indicated in the results refer to the corrected ones. Signal processing and statistical analyses were performed using MATLAB (version R2018a and R2022a Mathworks, Natick, MA), with customs functions.

## Results

### Clinical findings

We considered 257 patients that underwent SEEG over a period of 17 years; of these, 39 (17 females—44%) displayed both MED and MS preceded by at least 60 s of SEEG recording without any other pathological discharges. These 39 subjects were included in the study. We analyzed one MED and one MS for each patient. MEDs had a median duration of 3 s [interquartile range (IQR): 7; mean ± SD: 7.3 ± 13.3 s], whereas MS had a median duration of 25 s (IQR: 42.5; mean ± SD: 56.3 ± 74.5 s). Fragmentary rhythmic interictal spike bursts lacking frequency or spatial evolution were not considered MEDs. The median age at surgical treatment was 24 years (IQR: 18.5; mean ± SD: 24.5 ± 12.0 years), and the median epilepsy duration was 12 years (IQR: 14; mean ± SD: 14.6 ± 9.9 years). Structural lesions neuroradiologically identified and/or demonstrated after neuropathological evaluation were heterogeneous: seven focal cortical dysplasia (FCD) type I, five FCD type IIa, two FCD type IIb, six with no lesion at the MRI/histopathological examination, nine gliosis or scar, four periventricular nodular heterotopias, two cavernomas, two hippocampal sclerosis, one polymicrogyria with periventricular nodular heterotopia and one with oligodendroglial hyperplasia (MOGHE).

Post-surgical follow-up has a median of 74 months (IQR: 63.5; mean ± SD: 76.7 ± 41.8; range: 17–174). Post-surgical seizure outcome at the last control was Engel class I in 30 patients (76.9%) and Engel class II-IV in nine (23.1%). In all class II-IV patients, a poor surgical outcome are explained by incomplete resection of EZ due to its proximity to the eloquent cortex or to anatomical constraints. Clinical information is summarized in Table 1.

**Table 1:** Clinical findings

Patient	Sex	Age^a^	Epilepsy duration (years)^b^	Pathology (MRI and/or histopathological examination)	Follow-up (months)	Outcome^c^
1	M^d^	23	12	Cryptogenic	103	Ic
2	M	26	11	Cavernoma	106	IV
3	M	5	3	FCD^f^ IIa	174	III
4	F^e^	5	1.5	MOGHE	74	IV
5	M	12	8.5	FCD IIa	150	IV
6	F	44	38	Gliosis	76	Ia
7	M	35	9	Cavernoma	86	Ia
8	M	24	24	Cryptogenic	131	IV
9	M	7	1.5	Gliosis	17	III
10	M	19	5	Gliosis	38	Ia
11	M	20	10	Scar	27	Ib
12	M	23	13	FCD IIa	46	Ib
13	F	31	14	FCD IIa	98	Ia
14	F	39	11	Gliosis	108	Ia
15	F	31	19	PNH^g^	63	Ib
16	F	20	15	FCD (MRI)	80	Ia
17	M	26	25.5	FCD I	131	Ia
18	M	38	37	Hippocampal sclerosis	110	Ia
19	F	37	23	FCD IIb	60	Ia
20	F	14	8	FCD IIa	100	Ia
21	F	14	8	Gliosis	32	Ib
22	M	40	24	Cryptogenic (MRI)	45	Ib
23	F	34	20	FCD I	23	Ic
24	M	42	25	Scar	103	Ib
25	F	19	10	Cryptogenic	63	Ia
26	M	22	5	FCD Ia	84	II
27	M	37	27	FCD Ia	71	Ia
28	F	3	2.5	FCD I	161	Ib
29	M	28	15	Scar	19	IV
30	F	3.5	3	FCD Ib	133	IV
31	F	23	7	PMG^h^ + PNH	44	Ia
32	F	43	34	Hippocampal sclerosis	110	Ic
33	F	12	4	FCD Ib	61	Id
34	M	28	27	Cryptogenic (MRI)	80	Ia
35	M	7	5	PNH (MRI)	32	Ia
36	F	36	20	Cryptogenic (MRI)	62	Ic
37	M	27	16	Gliosis	29	Ic
38	M	23	12	PNH (MRI)	36	III
39	M	35	15	PNH	24	Ic

^a^Age: age at stereo-EEG (years).

^b^Epilepsy duration: years from the diagnosis to SEEG.

^c^Outcome: according to Engel’s classification.

^d^M, male.

^e^F, female.

^f^FCD, focal cortical dysplasia.

^g^PNH, periventricular nodular heterotopia.

^h^PMG, polymicrogyria.

The EZ in our patient cohort could involve one or more than one lobe, with a prevalence of the frontal (*n* = 27) and temporal (*n* = 17) lobes, followed by the parietal (*n* = 14), occipital (*n* = 7), and insular (*n* = 6) lobes.

### Static functional connectivity

#### Network global strength

**Figure 3:**
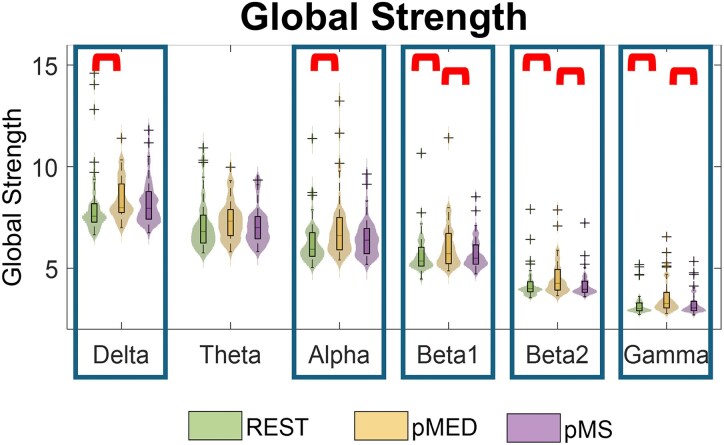
**Distribution plots of the global strength estimated from sFC.** Global strength was calculated across six frequency bands (Delta, Theta, Alpha, Beta-1, Beta-2, Gamma) and three conditions [REST, preceding Minor Electrical Discharge (pMED) and preceding Major Seizure (pMS)]. Each violin plot displays the full data distribution (*N* = 39), with the central horizontal line indicating the median, and the box width representing the IQR. Between-group statistically significant differences are marked with rectangles (*P* < 0.05, Friedman test FDR-corrected). *Post hoc* pairwise statistically significant differences are marked with red brackets (*P* < 0.05, Wilcoxon signed rank test FDR-corrected).

Figure 3 shows the distribution plots of the global strength in different SEEG frequency bands in the three different conditions (rest, pMED, and pMS). Statistical results for Friedman test and *post hoc* Wilcoxon signed-rank test are reported in Table 2. We found an interaction effect among the three conditions in all the frequency bands except for *theta*. For the same bands, *post hoc* comparisons indicated an overall increase of global strength during pMED compared with rest. Moreover, pMED global strength was also significantly higher than pMS for the *beta* and *gamma* bands. No differences were found between rest and pMS.

**Table 2:** Static functional connectivity: Statistical analysis of network global strength

		*δ*	*θ*	*α*	*β*1	*β*2	*γ*
**Friedman test**		*0.025*	0.129	*0.004*	*0.003*	*0.004*	*0.004*
***Post hoc* Wilcoxon signed rank**	rest-pMED	*0.044*	0.092	*0.012*	*0.017*	*0.000*	*0.000*
	rest-pMS	0.214	0.615	0.105	0.185	0.706	0.539
	pMED-pMS	0.567	0.092	0.105	*0.024*	*0.002*	*0.002*

Statistically significant differences are marked in italic (*P* < 0.05).

#### EZ and NEZ network topology

**Figure 4:**
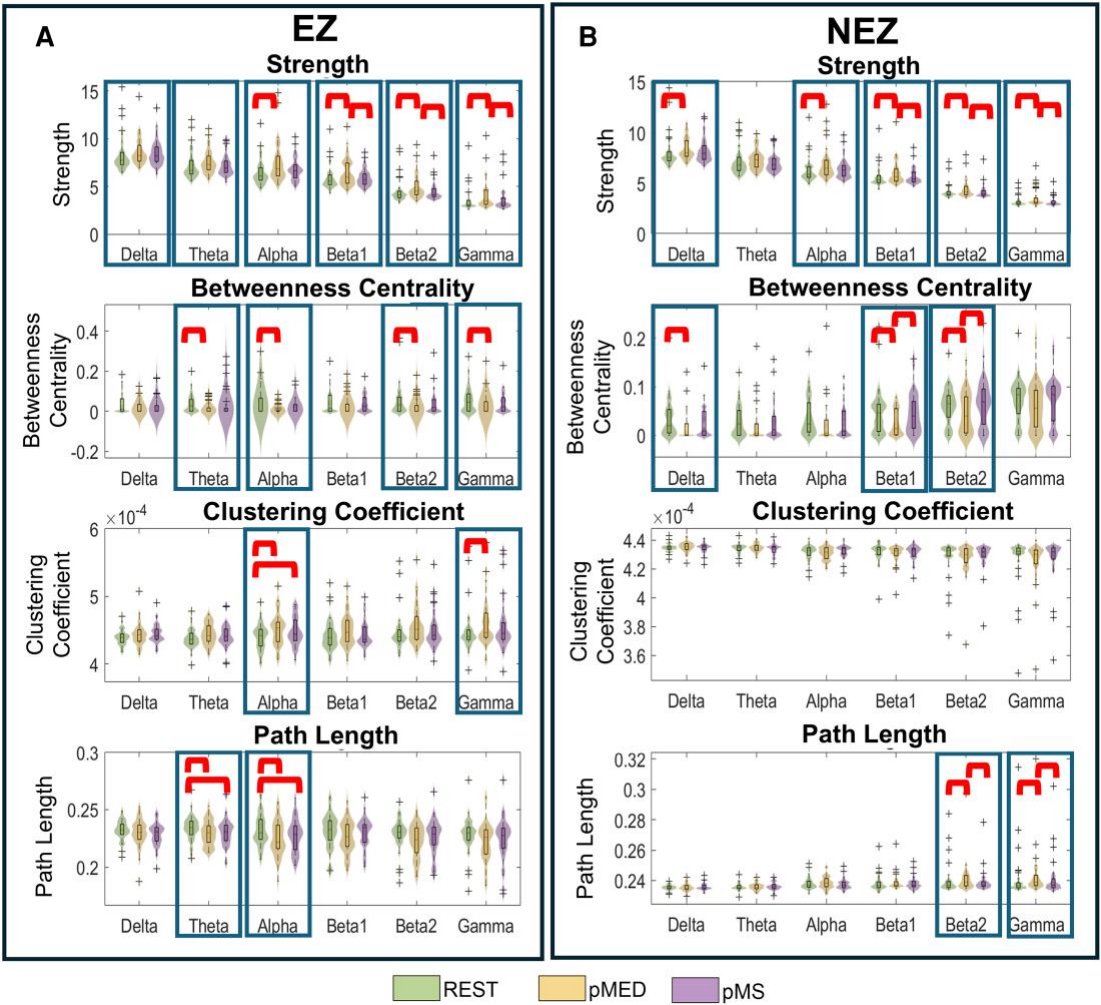
**Distribution plots of local sFC metrics.** Strength, betweenness centrality, clustering coefficient, and path length are shown (**A**) for the EZ and (**B**) for the NEZ regions, across six frequency bands (Delta, Theta, Alpha, Beta-1, Beta-2, Gamma) and three conditions [REST, preceding Minor Electrical Discharge (pMED) and preceding Major Seizure (pMS)]. Each violin plot displays the full data distribution (*N* = 39), with the central horizontal line indicating the median, and the box width representing the IQR. Statistically significant between-condition differences are marked with rectangles (*P* < 0.05, Friedman test FDR-corrected). *Post hoc* pairwise statistically significant differences are marked with red brackets (*P* < 0.05, Wilcoxon signed rank test, FDR-corrected).

The distribution plots and the statistical comparisons among conditions for all the four remaining sFC metrics are summarized in Fig. 4, for both the EZ (left panel) and NEZ (right panel). Statistical values for the Friedman test and *post hoc* Wilcoxon signed-rank test are reported in Table 3. In terms of nodal strength, Friedman test of EZ and NEZ showed similar results, with significant differences among the three conditions for all the frequency bands, except the NEZ *theta* band, with the most significant effect in the *beta2* and *gamma* for EZ. In the EZ, this difference was due to an increased nodal strength in pMED with respect to the rest in the *alpha* band, and increased values in pMED *beta1*, *beta2*, and *gamma* bands with respect to both rest and pMS conditions. The NEZ strength had a similar pattern, with the only exception of a significant lack of group interaction in the *theta* band.

**Table 3:** Static functional connectivity: Statistical analysis of local strength, betweenness centrality, clustering coefficient, path length in the EZ versus NEZ

	Friedman test											
	EZ						NEZ					
	*δ*	*θ*	*α*	*β*1	*β*2	*γ*	*δ*	*θ*	*α*	*β*1	*β*2	*γ*
**Strength**	*0.040*	*0.040*	*0.004*	*0.010*	*0.000*	*0.000*	*0.026*	0.113	*0.018*	*0.021*	*0.021*	*0.009*
**Betweenness Centrality**	0.195	*0.016*	*0.040*	0.074	*0.010*	*0.016*	0.021	0.438	0.201	*0.028*	*0.021*	0.168
**Clustering Coefficient**	0.074	0.074	*0.040*	0.202	0.074	*0.040*	0.724	0.601	0.828	0.327	0.113	0.314
**Path Length**	0.196	*0.040*	*0.040*	0.368	0.185	0.067	0.836	0.724	0.601	0.098	*0.021*	*0.018*

	*Post hoc* Wilcoxon signed rank											
	EZ						NEZ					
	*δ*	*θ*	*α*	*β*1	*β*2	*γ*	*δ*	*θ*	*α*	*β*1	*β*2	*γ*
	**Rest-pMED**						**Rest-pMED**					
**Strength**	0.057	0.069	*0.002*	*0.007*	*0.001*	*0.000*	*0.041*	0.095	*0.013*	*0.023*	*0.001*	*0.000*
**Betweenness centrality**	0.214	*0.011*	*0.005*	0.139	*0.004*	*0.020*	*0.000*	0.093	0.052	*0.019*	*0.014*	0.030
**Clustering coefficient**	0.220	0.062	*0.016*	0.157	0.039	*0.016*	0.615	0.780	0.503	0.335	0.067	0.098
**Path length**	0.387	*0.054*	*0.014*	0.125	0.051	0.042	0.748	0.615	0.558	0.162	*0.011*	*0.002*
	**REST-pMS**						**REST-pMS**					
**Strength**	0.101	0.435	0.062	0.214	0.264	0.264	0.251	0.645	0.115	0.209	0.834	0.635
**Betweenness centrality**	0.214	0.183	0.072	0.264	0.337	0.481	0.115	0.804	0.126	0.712	0.249	0.983
**Clustering coefficient**	0.042	0.095	*0.016*	0.655	0.176	0.209	0.696	0.674	0.645	0.823	0.655	0.460
**Path length**	0.062	*0.054*	*0.014*	0.485	0.128	0.180	0.748	0.615	0.867	0.738	0.645	0.410
	**pMED-pMS**						**pMED-pMS**					
**Strength**	0.485	0.108	0.091	*0.011*	*0.001*	*0.001*	0.503	0.095	0.115	*0.033*	*0.003*	*0.002*
**Betweenness centrality**	0.502	0.447	0.126	0.264	0.119	0.059	0.131	0.266	0.369	*0.019*	*0.013*	0.055
**Clustering coefficient**	0.225	0.686	0.258	0.238	0.069	0.118	0.615	0.754	0.326	0.569	0.044	0.098
**Path length**	0.284	0.780	0.350	0.306	0.128	0.180	0.748	0.933	0.558	0.415	*0.009*	*0.022*

Statistically significant differences are marked in italic (*P* < 0.05).

Regarding the EZ, betweenness centrality alterations involved the *theta*, *alpha*, *beta2*, and *gamma* bands, with a significant reduction in pMED compared with rest. Furthermore, changes in EZ segregation for *alpha* and *gamma* bands were observed, due to higher clustering coefficient in pMED with respect to the rest condition in both bands and higher pMS versus rest values in *alpha* band. Lastly, regarding the path length, we identified statistically significant differences in *theta* and *alpha* bands, due to a reduced pMED and pMS path length compared with rest.

NEZ betweenness centrality alterations involved the *delta*, *beta1*, and *beta2* bands, with a significant reduced value in pMED with respect to rest and pMS. Contrarily to the EZ, no statistically significant differences between the three conditions in terms of clustering coefficient were found. Lastly, differences in path length were statistically significant in *beta2* and *gamma* bands, with higher path length during pMED than during both rest and pMS conditions.

Overall, the results indicate the presence of network alterations differentiating pMED with respect to rest and pMS conditions, particularly in the *beta1*, *beta2*, and *gamma* bands, for all the topological properties (centrality, integration, and segregation). On the contrary, pMS showed a static network topology more like the rest condition, with only *theta* and *alpha* connectivity of the EZ indicating a network alteration during pMS with respect to the rest condition.

### Dynamic functional connectivity

**Figure 5:**
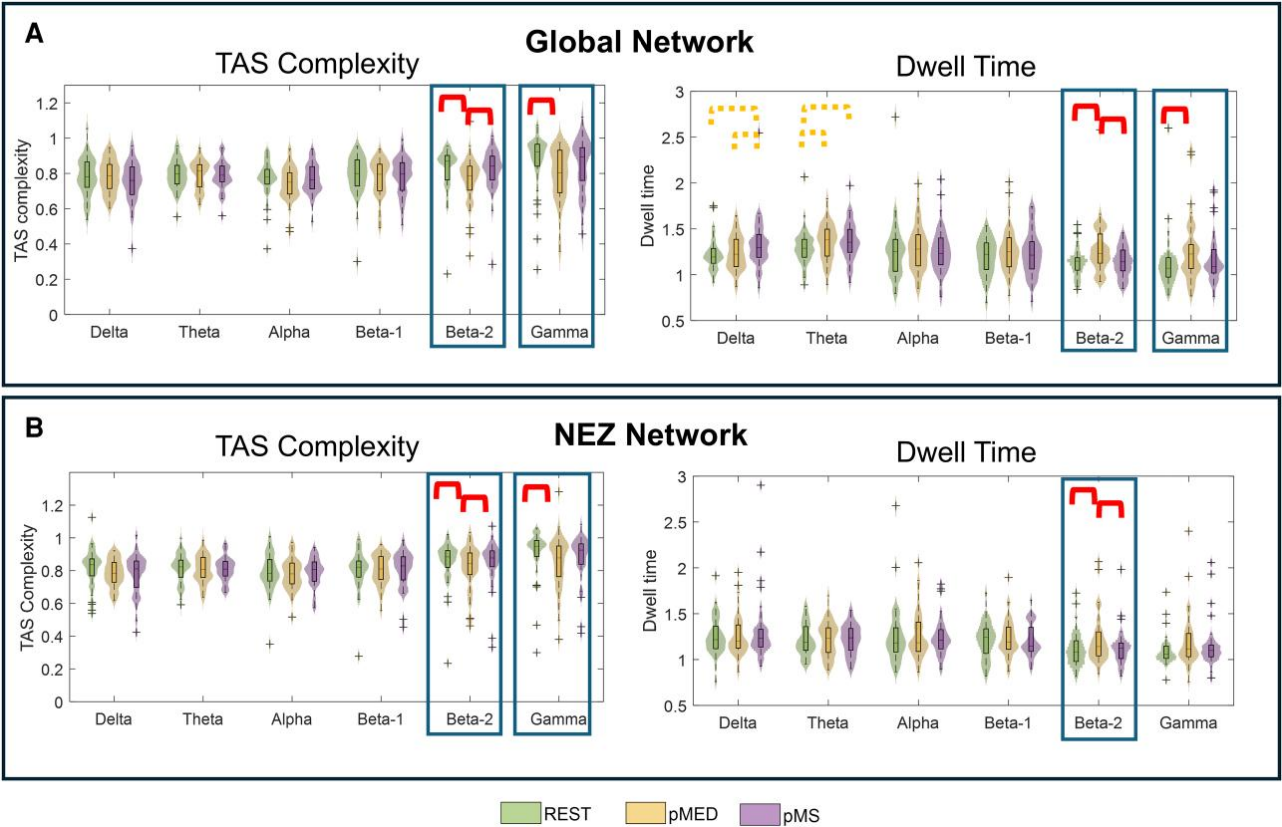
**Distribution plots of dFC metrics.** TAS complexity and dwell time are shown for (**A**) the global network and (**B**) the NEZ network, across six frequency bands (Delta, Theta, Alpha, Beta-1, Beta-2, Gamma) and three conditions [REST, preceding Minor Electrical Discharge (pMED) and preceding Major Seizure (pMS)]. Each violin plot displays the full data distribution (*N* = 39), with the central horizontal line indicating the median and the box width representing the IQR. Statistically significant between-condition differences are marked with rectangles (*P* < 0.05, Friedman test, FDR-corrected). *Post hoc* pairwise significant differences are indicated by red brackets (*P* < 0.05, Wilcoxon test, FDR-corrected). Orange dotted brackets indicate trends that did not reach significance after FDR correction but showed near-significant effects (*P* < 0.06).

To assess the dynamical patterns characterizing the three different conditions, two metrics of meta-state activation were calculated: TAS complexity and dwell time, both for the global network (including EZ and NEZ regions) and for the NEZ network only. The meta-states for the EZ subnetwork could not be estimated due to the low number of EZ contacts for each patient (ranging from 4 to 52), which made it impossible to obtain an accurate estimate of modularity. Figure 5 shows the distribution plots and the statistical comparisons between conditions for TAS complexity (left panel) and dwell time (right panel), calculated on the global network (Fig. 5A) and on the NEZ subnetwork (Fig. 5A). Statistical values for the Friedman test and *post hoc* Wilcoxon signed-rank test are reported in Table 4. The overall results reveal an altered dFC in the *beta2* and *gamma* frequency bands for the pMED condition compared with both rest and pMS, primarily driven by NEZ subnetwork. This alteration is characterized by lower TAS complexity and higher dwell time in pMED relative to the other two conditions, indicating reduced dynamical variability in the temporal reorganization of network topology and stability during this state.

**Table 4:** Statistical analysis of Dwell time and TAS complexity in the whole network versus the NEZ subnetwork

		Dwell time											
		Whole network						NEZ subnetwork					
		*δ*	*θ*	*α*	*β*1	*β*2	*γ*	*δ*	*θ*	*α*	*β*1	*β*2	*γ*
Friedman test		0.175	0.116	0.174	0.199	*0.008*	*0.035*	0.926	0.882	0.882	0.664	*0.022*	0.174
*Post hoc* Wilcoxon signed rank	rest-pMED	0.780	0.067	0.308	0.316	*0.008*	*0.007*	0.738	0.748	0.615	0.592	*0.022*	*0.026*
	rest-pMS	0.069	0.067	0.329	0.664	0.717	0.315	0.738	0.748	0.615	0.791	0.878	0.199
	pMED-pMS	0.069	0.834	0.738	0.738	*0.008*	0.314	0.738	0.748	0.615	0.592	*0.022*	0.145

		TAS complexity											
		Whole network						NEZ subnetwork					
		*δ*	*θ*	*α*	*β*1	*β*2	*γ*	*δ*	*θ*	*α*	*β*1	*β*2	*γ*
Friedman test		0.700	0.498	0.251	0.794	*0.004*	*0.046*	0.836	0.836	0.285	0.552	*0.034*	*0.005*
*Post hoc* Wilcoxon signed rank	rest-pMED	0.823	0.856	0.125	0.748	*0.008*	*0.006*	0.501	0.878	0.664	0.845	*0.053*	*0.008*
	rest-pMS	0.823	0.856	0.606	0.748	0.878	0.402	0.592	0.878	0.706	0.845	0.834	0.199
	pMED-pMS	0.583	0.856	0.321	0.748	*0.017*	0.172	0.856	0.878	0.664	0.845	*0.026*	0.092

Statistically significant differences are marked in italic (*P* < 0.05).

#### Global network (EZ + NEZ)

A significant interaction between conditions was found in the *beta2* and *gamma* bands for both TAS complexity and average dwell time. *Post hoc* comparisons indicated statistically significant lower values of TAS complexity for pMED compared with rest. On the other hand, the *post hoc* analysis of dwell time metric indicated an increased dwell time for pMED compared with rest in the same frequency bands. For both metrics, no significant differences between rest and pMS were found. However, pMS was characterized by higher dwell time than rest and pMED in the *delta* and *theta* frequency bands, with differences close to statistical significance (*P* = 0.06). Consistently with sFC results, lower frequency ranges appear to be associated with FC alterations leading to MS, while higher frequency being related to specific alterations characterizing pMED condition. This difference is not statistically significant after FDR correction, whereas showed a trend toward this direction.

#### NEZ network

Considering only the NEZ subnetwork, the dFC results revealed the same pattern of differences than for the global network in the *beta2* and *gamma* band FC in pMED with respect to rest and pMS, with a reduced TAS complexity and higher dwell time in pMED. On the other hand, after removing the EZ, the trend of longer dwell time during pMS in the *delta* and *theta* range disappeared, suggesting a more specific role of the EZ low-frequency functional connectivity in seizure dynamics. Of note, the statistically significant differences in dwell time in *gamma* also disappeared if the EZ was not considered.

### Correlation between sFC and dFC metrics

To assess the extent of correlation between sFC and dFC results we quantified the Pearson’s correlation between dFC meta-state activation measures—specifically average dwell time and TAS complexity—and sFC graph metrics (global strength, NEZ strength, betweenness centrality, clustering coefficient and path length) in the NEZ. Analyses were conducted across the three conditions of interest (rest, pMED, pMS) and two frequency bands (beta and gamma) selected based on prior findings of significant alterations. Results are summarized in Table 5, with statistically significant correlations (*P* < 0.05) highlighted in red and bold.

**Table 5:** Pearson correlations of the sFC graph measures averaged over electrodes with the DFC meta-state activation measures in the beta (upper table) and gamma (lower table) bands

*β*	Global strength			Strength NEZ^a^			Betweenness centrality NEZ			Clustering coefficient NEZ			Path length NEZ		
	Rest	pMED^b^	pMS^c^	Rest	pMED	pMS	Rest	pMED	pMS	Rest	pMED	pMS	Rest	pMED	pMS
Average dwell time	*0.83*	*0.45*	0.17	*0.82*	*0.49*	0.2	*−0.39*	*−0.37*	−0.17	*−0.34*	0.13	0.1	*0.46*	−0.08	−0.07
TAS^d^ complexity	*−0.9*	*−0.55*	−0.29	*−0.87*	*−0.56*	0.29	*0.51*	*0.48*	0.14	*0.49*	0.07	0.1	*−0.59*	−0.11	−0.12

γ	Global strength			Strength NEZ			Betweenness centrality NEZ			Clustering coefficient NEZ			Path length NEZ		
	Rest	pMED	pMS	Rest	pMED	pMS	Rest	pMED	pMS	Rest	pMED	pMS	Rest	pMED	pMS
Average dwell time	*0.68*	*0.71*	*0.44*	*0.56*	*0.75*	*0.51*	*−0.43*	*−0.52*	*−0.41*	*−0.62*	−0.05	−0.08	*0.62*	0.15	0.08
TAS complexity	*−0.83*	*−0.74*	*−0.55*	*−0.68*	*−0.72*	*−0.55*	*0.51*	*0.6*	*0.45*	*0.77*	0.27	*0.32*	*−0.78*	*−0.35*	*−0.32*

Statistically significant correlations (*P* < 0.05) are marked in italics.

^a^NEZ, non-epileptogenic zone.

^b^pMED, pre-minor electrical discharge.

^c^pMS, pre-major seizure.

^d^TAS, time activation sequence.

Across both frequency bands, the rest condition consistently showed significant correlations between all the sFC and dFC metrics. Specifically, global strength, local strength and path length were positively correlated with average dwell Time and negative correlated with TAS complexity, indicating that stronger network integration is associated with longer persistence in meta-states and more stable dynamic. In contrast, betweenness centrality and clustering coefficient were negative correlated with dwell time and positive correlated with TAS complexity, suggesting that greater network segregation corresponds to more variable and complex dynamic configurations.

In contrast to rest, pMED and pMS conditions revealed distinct and frequency-dependent correlation profiles. Interestingly, in the beta band, pMS showed no significant correlations with any sFC metric, suggesting a complete decoupling between static network topology and dynamic behaviour. pMED retained significant correlations, with global strength, strength NEZ, and betweenness centrality, but not with clustering coefficient or path length, indicating preserved integration and centrality-related correlation with dFC, but altered segregation-related associations.

In the gamma band, both pMED and pMS maintained significant correlations across most dFC and sFC metrics, except for the absence of significant correlation between dwell time and clustering coefficient and path length.

These findings reveal a condition- and frequency-specific modulation of the relationship between static and dynamic connectivity. While rest consistently reflects strong correlation between sFC and dFC, pMED preserves this relationship selectively—especially in the gamma band—whereas pMS disrupts it entirely in the beta band.

## Discussion

As reflected by the extensive literature in the field, epilepsy research has progressively shifted from a local perspective toward a dynamic, complex, and global network framework, revealing that the epileptogenic brain network undergoes topological and functional alterations across interictal, preictal, and ictal stages.^60-62^ Understanding the dynamics of epileptogenic networks is of great clinical relevance, as it is essential for elucidating the mechanisms underlying seizure generation and propagation. However, despite this progress, very few studies have examined the fine-grained temporal reorganization of intracerebral networks immediately preceding different types of epileptic events. In particular, the transient and subclinical nature of MED has often led them to be overlooked, leaving unexplored whether they share—or diverge from—the mechanisms that drive major seizures (MS).

**Figure 6:**
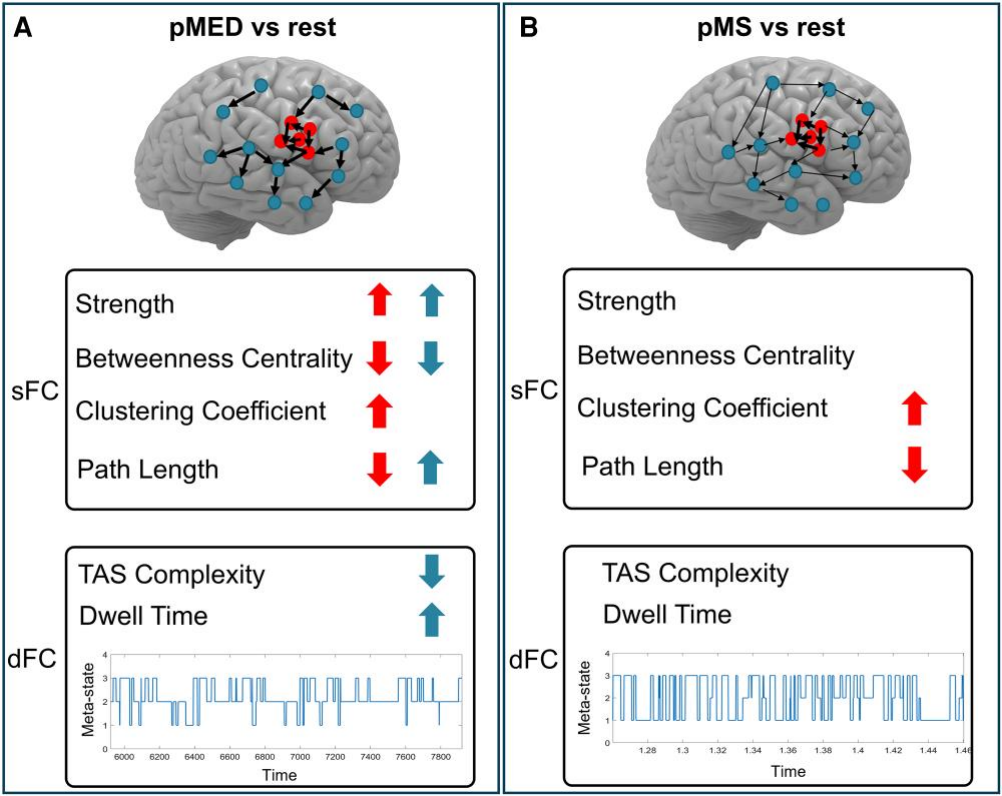
**Schematic representation of key findings from static (sFC) and dynamic (dFC) functional connectivity analyses.** Left panel (**A**): network changes between REST and preceding Minor Electrical Discharge (pMED); right panel (**B**): network changes between REST and preceding Major Seizure (pMS). Red blobs indicate nodes within the EZ, while blue blobs represent nodes in the NEZ. Thicker black arrows mark statistically significant differences between conditions. Within the sFC and dFC quadrants, red and blue arrows denote significant increases (upward) or decreases (downward) in connectivity strength. The lower plots display Temporal Activity Structure (TAS) sequences derived from dFC analysis for a representative patient, revealing a more stable network configuration during pMED compared with the more unstable patterns observed in REST and pMS. Overall, the figure demonstrates that both sFC and dFC metrics capture a distinct network reorganization during pMED, with a prominent role of the NEZ. This shift suggests a protective configuration that may help prevent escalation into a major seizure event.

In this study, we introduce a novel framework combining static and DFC analyses of SEEG data to differentiate the network alterations that precede MED and MS onset in patients with drug-resistant epilepsy. By integrating graph-theoretical characterization of static topology with the identification of recurrent dynamic connectivity states, our approach provides an unprecedented view of how large-scale epileptogenic networks reorganize in time before seizure expression. We hypothesized the presence of a protective mechanism involving both the EZ and the NEZ, capable of preventing the epileptogenic network from evolving into a major seizure. Our findings provide the first direct evidence of distinct preictal network configurations associated with MED and MS, supporting the existence of inhibitory or compensatory processes that act beyond the EZ to contain abnormal activity. Our results revealed distinct patterns of sFC and dFC preceding MED compared with both resting state and MS, mainly involving the NEZ. In line with previous evidence of antagonistic mechanisms that can enhance or suppress seizures,^29,30^ these results suggest the existence of potential protective mechanisms, involving brain regions beyond the EZ, that may prevent the full emergence of a major seizure, confining the activity to a minor electrical discharge. A comprehensive overview of the main findings about both sFC and dFC is represented in Fig. 6.

### SFC analysis

Our analysis revealed significant alterations in network centrality, integration, and segregation properties, involving both the EZ and NEZ. Specifically, we identified an overall increased strength and a reduced betweenness centrality during pMED compared with resting state and pMS, particularly in the *beta* and *gamma* frequency bands, for both EZ and NEZ sub-networks. Strength and betweenness centrality are two complementary measures used to characterize the importance or centrality of nodes within a network. Strength quantifies the overall connectivity of a node in terms of number and strength of connections with other nodes, while betweenness centrality quantifies the extent to which a node acts as a bridge between different parts of the network.^41^ Their opposite trends (increased strength and reduced betweenness) suggest that the network’s nodes gain stronger local connections, while reducing their role as crucial bridges connecting different parts of the network. This scenario suggests a protective shift in the network structure, occurring only during pMED (and not during pMS), where nodes become more locally interconnected while reducing their role of critical hubs as connectors, thus being less sensitive to disruptions in specific regions. On the other side, the analysis of path length and clustering coefficient (measures of integration and segregation, respectively) revealed a different behaviour between the EZ and NEZ regions. The EZ shows a reduced path length and an increased clustering coefficient during both pMED and pMS compared with the rest condition. This reflects a more integrated network within the EZ, with higher modular organization and intragroup connections, while reducing the interactions with other regions. In contrast, the NEZ network exhibited a significant increase in path length during pMED, but not during pMS. This finding suggests that the NEZ, by transiently reducing its integration with other brain regions specifically during pMED events, may play an inhibitory role in seizure propagation, thereby controlling the progression from MED to MS. Altered integration and segregation properties have been extensively associated with brain network in epilepsy. In a comprehensive review with meta-analysis, Slinger and colleagues^63^ reported that interictal structural networks of epileptic patients have a lower level of integration with respect to healthy controls. Previous studies also pointed out higher levels of segregation in epileptic patients, although this result is less homogeneous among studies.^63,64^ Our results highlight the distinct and opposite role of the EZ and NEZ in modulating integration and segregation prior to epileptic activity. Specifically, in the EZ, we observed increased integration and reduced segregation for both pMED and pMS, which may serve as mechanisms that predispose the network to seizure initiation. Conversely, the NEZ reduces network integration exclusively during pMED, but not during pMS, suggesting its role in suppressing the evolution of a MED into a MS.

While the primary aim of this study is not to localize the EZ and differentiate it from NEZ, our results of sFC indicate differences between EZ and NEZ metrics, specifically for the path length and clustering coefficient. These findings suggest that static connectivity metrics may also hold promise as a complementary tool for supporting EZ identification.

Static connectivity alterations in epileptogenic networks have been widely studied,^15,65^ with inconsistent results possibly due to differences in methodological approaches as well as to the considerable localization or etiology variability in focal epilepsies, and the different criteria utilized to define the EZ. Most evidence suggests that resting state networks in focal DREs are associated with increased local connectivity in the EZ and decreased connectivity in widespread distant brain regions,^65^ with the EZ characterized by higher inward connectivity during resting state.^66,67^ These findings were confirmed by cluster partitioning analysis of intracerebral field responses evoked by intracerebral stimulation during SEEG,^68^ that demonstrated strong bidirectional connectivity between contacts included in the EZ. While epileptic networks have been largely associated with hyper-synchronization of brain activity, models of more complex interactions among hyper-synchronized and desynchronized sub-network nodes are being increasingly associated with seizure dynamics.^69-71^

Previous SEEG studies have underlined the relevance of connectivity features not only in EZ but also in the surrounding regions. Specifically, Lagarde *et al*.^72^ demonstrated that interictal functional connectivity is hierarchically organized, being gradually enhanced within the EZ and the propagation zone. Crucially, they found that higher functional connectivity within the non-involved zone was associated with a poorer post-surgical outcome, suggesting that a more distributed functional network carries prognostic value. In a complementary approach, Besson *et al*.^73^ investigated structural connectivity, observing that structural deterioration does not primarily target the EZ propagation zone core, but rather manifests as a distributed network disintegration affecting large-scale functional systems such as the salience network and the default mode network. These findings collectively highlight the need to move beyond the EZ delineation by considering both the intrinsic connectivity of the epileptogenic regions and the role of distributed, seemingly non-epileptogenic networks in modulating epileptogenesis and surgical success.

Recent studies propose the existence of inhibitory mechanisms that actively involve peri-EZ healthy brains regions that prevent the EZ from starting seizures. In a recent study, Johnson and colleagues^74^ postulated the interictal suppression hypothesis, a mechanism of seizure suppression in which the seizure onset zones are actively segregated by other healthy regions of the brain, suppressing their ability to connect with the rest of the network and initiate a seizure. The same authors reported a behaviour of EZ during intracerebral stimulation, characterized by a relative decrease of *theta* band power coupled with a relative increase of *beta/gamma* band power when NEZs were stimulated.^74^ It has been long known that *theta* power is involved with long-range integration of brain regions, whereas *gamma* power is crucial for local integration.^75^ Thus, these findings suggest that EZ functional segregation is increased when NEZs are stimulated, underlying direct influence of NEZ on EZ.^74^ Accordingly, our results confirm statistically significant differences in EZ clustering coefficient in the *theta-alpha* range in the rest condition with respect to pMS and pMED, supporting the hypothesis of a higher EZ segregation before seizure onset.

Several studies have provided evidence supporting the *interictal suppression hypothesis*, which attributes to regions beyond the EZ a critical role in inhibiting EZ activity during the interictal period, and suggests that seizures occur when this inhibitory mechanism fails.^27,28,74^ In a recent work, Karimi-Rouzbahani and McGonigal,^26^ further demonstrated that the EZ predominantly receives neural input from NEZ in between seizures, while mainly transmits neural activities to NEZ during seizures, consistent with a suppressive influence aimed at preventing seizure generation. Narasimhan *et al*.,^67^ highlighted high inward connectivity in the EZ reflecting inhibitory input from other regions to prevent seizure activity, which may flip the direction when seizure activity begins. Similarly, Gunnarsdottir and colleagues^28^ and developed a ‘source-sink’ metric to accurately identify the EZ, based on the hypothesis that when a patient is not having a seizure, the EZ ‘sink’ nodes is inhibited by neighboring regions (the ‘source’ nodes). Our theoretical scenario shows an EZ similarly organized (and thus, similarly prone to generating seizures) in both conditions (pMEd and pMS), while NEZ is organized differently in pMED and pMS. Our findings appear coherent with the previous hypothesis’ but using a novel model to discuss the differential capacity of the brain to suppress or permit seizures before MS and MED (not only during the rest).

### DFC analysis

DFC analysis allowed us to explore the temporal evolution of brain FC meta-state organization preceding MED and MS onset. Meta-states analyses revealed distinct temporal patterns of network activation during different conditions, with lower TAS complexity and longer dwell time observed during pMED compared with both rest and pMS conditions, particularly in high frequencies activity bands. The reduction in TAS complexity suggests a more constrained and less variable network state preceding MED onset. Furthermore, longer dwell times during pMED indicate longer period of persistence in meta-states, reflecting prolonged periods of network stability. This pattern did not vary when considering either the global network or the sub-network composed only by the NEZ. Overall, our results suggest a protective mechanism activated before MED, based on the reduction of the intrinsic dynamic network fluctuation that characterizes epileptogenic networks. Interestingly, meta-state dFC analysis did not reveal a different pattern preceding seizures (i.e. in pMS), except for a trend characterizing the EZ role in low-frequency ranges to increase the stability of meta-states (longer dwell time) before seizure onset. This could suggest an opposite mechanism facilitating seizures. However, the method applied, based on similarity recurrence plots, does not allow to properly studying the specific EZ dynamics, due to the low number of nodes composing this sub-network preventing a reliable estimation of network similarity across time.

Dynamic FC has been recently developed and employed to characterize the alterations that schizophrenia, mild cognitive impairment, and Alzheimer’s disease elicit in meta-states topology and their dynamical transitions in auditory oddball task and resting state scalp EEG recordings.^7,8^ The study of dynamic epileptic networks is relatively recent and most of the studies in literature are based on fMRI data, which cannot provide information about fast temporal neural oscillations. In this framework, some recent studies suggested that epileptic patients have higher fluctuation of resting state FC over time compared with healthy controls,^76,77^ thus suggesting that temporal stability/instability of network organization is a useful biomarker of ictogenesis. Khambhati *et al*.^78^ defined different brain statuses, associated with different connectivity configuration during pre-ictal, ictal, and post-ictal stages. Their results showed that epileptic networks are characterized by hubs of persistent strong connections surrounded by rapidly reconfiguring weak connections that, preceding seizure generation, benefit from high network flexibility to drive seizure generation through a rapid reorganization of weaker connections in the epileptic network.^78^ In a further study, the same team proposed a ‘push-pull control’ mechanism on virtually resected epileptic brains.^29^ According to this model, desynchronizing and synchronizing nodes act as antagonists’ determinants of the spread—or confinement—of dynamic processes. Starting from electrocorticography data, the authors demonstrated that synchronizability in the high-*gamma* networks is increased before seizures that spread compared with seizures that remain focal. Also, with an innovative method of virtual cortical resection, they identified synchronizing and desynchronizing brain regions. These control regions act differently in the pre-seizure and seizures epochs, especially in the area surrounding the EZ. In line with this evidence, our overall results suggest a protective mechanism based on the temporal reorganization of network topology, especially the NEZ, into a less flexible and less dynamically variable network able to influence (or even prevent) seizure occurrence.

Our key findings of a main role of higher frequencies (beta/gamma) in the protective functional sFC and dFC changes during pMED events aligns strongly with the literature linking beta/gamma rhythmogenesis to perisomatic inhibition mediated by GABAergic interneurons.^79^ This high-frequency activity maintains cortical networks in a moderately active state that is robust against massive neuronal recruitment, preventing the transition to a full seizure. The dFC stability and reduced complexity observed during pMED thus represent the macroscopic signature of this enhanced inhibitory control. Furthermore, the ability of the network to achieve this coordinated, stable state is likely facilitated by mechanisms of temporal synchronization. Dickson and de Curtis^80^ demonstrated that synaptic activation can induce a phase reset of ongoing gamma activity, leading to a transient enhancement of synchronization across distant cortical sites. This mechanism provides a way for the NEZ to exert a likely unified controlling influence over the EZ. In this context, the pMED dFC enhanced stability reflects a successful network-level defense against seizure propagation, whereas its absence prior to MS suggests that the underlying ictal activity either rapidly overwhelms this dynamic barrier or that the barrier fails to engage effectively.

### Correlation between sFC and dFC

The analysis of the correlation between sFC and dFC metrics revealed a different dissociation between static and dynamic connectivity across conditions and frequency bands, and provides insight into how network architecture supports or constrains brain dynamics, differentially in rest than preceding epileptic events.

The relationship between sFC and dFC is an interesting current topic of research, whereas this relationship has not been yet fully understood, especially in pathological alterations of brain network organization. Previous studies showed that whereas static and DFC capture overlapping aspects of brain network organization, dFC captures additional relevant variability linked to behavioural or cognitive state.^2,81^ In epilepsy research, the interplay between sFC and dFC has gained increasing attention as could offer novel insight into how brain networks reorganize during interictal pre-ictal and ictal states. Previous studies have shown that dFC may offer greater sensitivity to understand pathological network changes in epilepsy than sFC. For example, Pang *et al*.^82^ found that while both sFC and dFC were altered in temporal lobe epilepsy, dFC measures like variability and dwell time were more closely linked to disease severity and cognitive impairment. Similarly, Zhang *et al*.^83^ reported that in generalized epilepsy, static hyper-connectivity coexisted with reduced dynamic flexibility, suggesting that sFC may obscure underlying network rigidity. Additionally, Preti *et al*.^84^ demonstrated that dFC can reveal transient disruptions in epileptic networks—particularly during pre-ictal and interictal phases—that are not captured by static analysis, highlighting its potential as a biomarker for seizure prediction and localization.

In line with these previous evidences, the condition-specific differences in the correlation between sFC and dFC observed in our data support the notion that dFC provides additional insights into the pathological organization of epileptic networks The consistent correlations at rest suggest that both static integration and segregation properties of the network are tightly coupled with dynamic meta-state behaviour, supporting stable and structured transitions. In contrast, the absence of significant correlations between sFC and dFC during pMS—compared with the partially preserved correlations during pMED—suggests that transient, time-varying reorganization of the network may play a crucial role in seizure generation. Although investigating the relationship between sFC and dFC within the epileptogenic network falls outside the primary scope of our study, these findings indicate that changes in dFC between interictal and ictal conditions could reveal aspects of network dynamics that static connectivity analyses alone cannot fully capture.

### Limitations and future research lines

This study has several limitations that warrant discussion. Regarding the meta-state analysis, a deeper understanding of the specific impact of the EZ on state dynamics would be valuable. However, in some subjects, the limited number of SEEG electrodes prevented accurate estimation of functional connectivity (FC) network self-similarity when focusing solely on EZ contacts, thereby hindering the application of the Louvain algorithm for community detection. Addressing this challenge could involve methodological advancements, such as performing source estimation on the SEEG data to create a full cortical source map of SEEG activations. Additionally, while the average dwell times and TAS complexity provides a broad overview of meta-state dynamics, future research could delve into the specific timing of state activations. Such an approach could facilitate a more detailed per-subject analysis of state dynamics and their potential connection to inhibitory mechanisms that might prevent seizures in the pMED condition compared with pMS. The intrinsic differences in network size across subjects in sEEG constitute another inherent limitation of this study and of the data itself, as they may affect the comparability of network metrics and the interpretation of connectivity patterns. Although the applied strategies mitigate the influence of the network size, they cannot fully eliminate it. Nevertheless, our analyses focused on within-subject comparisons across three conditions (rest, pMED, pMS). Since the network size for each subject remained constant across conditions, this design also helps to reduce the potential bias introduced by inter-subject variability. Future studies should consider complementary techniques with more homogeneous brain coverage, such as EEG or MEG, to enable analyses of networks with consistent size across participants.

The choice of the connectivity metric used to estimate sFC and dFC is another important factor that could influence both the results and the interpretation of the underlying mechanism. A recent study compared different 15 FC metrics in intracranial EEG recordings, showing that they differ in their ability to describe changes in the EZ network before and during seizures, as well as in their capacity to identify the EZ itself.^26^ While there is a wide range of methods available for estimating sFC, in our study the choice was constrained by the requirement of having both static and dynamic versions of the same method. Although we focused on amplitude-based connectivity metrics, it is known that phase-based connectivity approaches can provide complementary, non-redundant information that may reflect distinct neurophysiological mechanisms. Amplitude coupling may arise from slow, global processes such as neuromodulation or extracellular ion fluctuations, whereas phase coupling is often driven by synaptic interactions and sensory processing.^85,86^ Extend this study including phase-based connectivity analysis—such as Phase Locking Value (PLV)^87^ for sFC and Phase Difference Distribution (PDD)^86^ for dFC—would be a promising direction for future research, potentially offering a more comprehensive view of the role of network dynamics in seizure generation or inhibition.

Another limitation is that this study focused only on the period preceding MED and MS, without analyzing seizure progression. This constraint arose because the dFC method employed requires consistent time-window comparisons across events of interest, and MED and MS differ in duration. Future research could explore alternative approaches to analyze seizure progression dynamics despite these differences and hypothetically correlate seizure severity with the pre-ictal and propagation network. Furthermore, while temporal resolution is excellent, SEEG shows limited spatial resolution and implantation is patient-dependent. It must also be considered that our evaluation is aimed at patients with focal seizures, nothing can currently be inferred about generalized epilepsies which represent another interesting population to be evaluated in future studies. Finally, the relationship between sFC and dFC results and the underlying pathology was not analyzed, nor was the effect of antiseizure medications used by the patients during recordings. We decided to analyze a single MED and MS event for each patient to keep certain variables constant: wakefulness status and medication dosage. In addition, we had to discard MED and MS that had ictal discharges in the previous 60 s, thus significantly limiting the number of events available. Future prospective studies with a larger dataset could verify the reproducibility of inter- and intra-patient results, as well as correlate network’s changes with clinical findings

## Conclusions

This study explored the differences in brain networks preceding pMED and pMS in patients with drug-resistant epilepsy, using a comprehensive approach that combines static and DFC. Our findings highlight distinct patterns of brain activity that precede pMED compared with resting state and pMS, suggesting the existence of inhibitory mechanisms involving NEZ capable of suppressing the onset and spread of seizures from EZ.

Overall, our results suggest that the brain network structure dynamically reconfigures to either hinder or facilitate seizures. The NEZ, in particular, appears to play a crucial protective role by stabilizing the network and preventing the spread of seizures. These insights pave the way to better understanding and treatment of epilepsy by targeting these dynamic network changes.

## Data Availability

The data supporting the findings of this study are not publicly available due to privacy restrictions, and are available from the corresponding author upon reasonable request. The custom scripts used to compute the sFC analysis are available at (https://github.com/giuliavarotto/sFC_Epilepsy). The implementation for calculating the IAC can be accessed on GitHub (https://github.com/Prejaas/High-temporal-resolution-MEG-measures-of-functional-connectivity). Additionally, the code supporting the computation of data-driven windows, meta-state extraction, TAS and ICT, as well as the metrics related to meta- state activation, is also hosted on GitHub (https://github.com/pablonuneznovo/Meta-state-extraction-from-high-temporal-resolution-connectivity).
